# Novel Preparation of Reduced Graphene Oxide–Silver Complex using an Electrical Spark Discharge Method

**DOI:** 10.3390/nano9070979

**Published:** 2019-07-05

**Authors:** Kuo-Hsiung Tseng, Hsueh-Chien Ku, Der-Chi Tien, Leszek Stobinski

**Affiliations:** 1 Department of Electrical Engineering, National Taipei University of Technology, Taipei 10608, Taiwan; 2 Materials Chemistry, Warsaw University of Technology, Warynskiego 1, 00-645 Warsaw, Poland

**Keywords:** graphene oxide, reduced graphene oxide, silver nanoparticle, electrical spark discharge method, nanocomposite

## Abstract

This study used an electrical discharge machine (EDM) to perform an electrical spark discharge method (ESDM), which is a new approach for reducing graphene oxide (GO) at normal temperature and pressure, without using chemical substances. A silver (Ag) electrode generates high temperature and high energy during gap discharge. Ag atoms and Ag nanoparticles (AgNP) are suspended in GO, and ionization generates charged Ag^+^ ions in the Ag plasma with a strong reducing property, thereby carrying O away from GO. A large flake-like structure of GO was simultaneously pyrolyzed to a small flake-like structure of reduced graphene oxide (rGO). When Ag was used as an electrode, GO was reduced to rGO and the exfoliated AgNP surface was coated with rGO, thus forming an rGOAg complex. Consequently, suspensibility and dispersion were enhanced.

## 1. Introduction

Graphene consists of two-dimensional hexagonally arranged carbon atoms having a thickness of one atom. It can be used for applications in energy storage [1] and biotechnology [2] because of its excellent mechanical [3], electronic [4], and chemical properties [5,6]. Graphene is used in molecular sensors [7], supercapacitors, [8] and bacteria repellency [9]; therefore, its preparation must be further studied. Since graphene is difficult to produce, we employed the graphene oxide (GO) reduction method to prepare reduced graphene oxide (rGO), which featured physical properties closest to those of graphene. Many chemical synthesis methods are currently available, and the reduction method is prevalently used for the mass production of graphene. The reduction method can reduce GO to rGO, and it is currently the most efficient, rapid, and affirmative practice [10].

Since GO has no electrical or thermal conductivity, it is often reduced to rGO to recover partial electrical and thermal conductivities. Different methods are currently available for reducing GO to rGO, and chemical reduction is the most recognized method. Hydrazine [11,12], NaOH [13,14], NaBH_4_, [15,16], and dimethylhydrazine [17] are the commonly used reducers. However, reduction with these reducers has some limitations, as the process is time consuming and highly complex, and the reducer is also hazardous, that is, it can be harmful if touched by accident and it can pollute the environment if inappropriately disposed. To avoid these problems, some studies have used different reducers for reduction, including aluminum powder [18], vitamin C [19], protein [20], and sodium citrate [21]; which are considered environment friendly and safe. Nonchemical reduction methods, such as electrochemical reduction [22,23,24], thermal reduction [25], the microwave method [26], and the plasma-assisted method [27], are also available. For reduction, these methods primarily use the energy derived from different sources and from physical and chemical changes.

The current study aimed to develop a simple, rapid, and environment-friendly method, where GO is reduced to an rGO complex through the electrical spark discharge method (ESDM) [28] using an electrical discharge machine (EDM) and a silver electrode. The underlying principle of this method is that when the silver electrode discharges in GO dispersion, high energy is generated. This energy melts the solid silver electrode, thus forming liquid silver (Ag). The high temperature vaporizes liquid Ag into Ag atoms, which are ionized by a high field of 25–50 KV/cm to form plasma [29]. Then, the Ag atom loses electrons and generates Ag^+^. Since the Ag plasma has a strong reducing property, the epoxide functional group (O^2−^) of GO is generally reduced during discharge, and GO is then reduced to rGO. This preparation requires an EDM, GO, silver wires, and a magnetic stirrer. The EDM is the most crucial equipment. GO is a dielectric fluid, and silver wire is used as an electrode. Furthermore, the magnetic stirrer disperses Ag metal particles stripped by an arc [30], thus maintaining the insulativity of the dielectric fluid. A conventional EDM is generally used for cutting techniques and perforation processes. A considerable amount of successful research is available on such techniques [31,32,33]. The ESDM is a physical and not a chemical method, and it is simpler, faster, and easier for collecting nanometal colloids than conventional methods. This method can be performed at normal temperatures and pressure. In this method, the water-soluble GO is reduced using the EDM with silver electrode discharge. This reduction process is simple and time efficient and rGO covers Ag to form an rGOAg complex colloid. Since the molecular structure of rGO retains other functional groups, it is advantageous for bonding and dispersion in other substrates, indicating that it is suitable for the preparation of composite materials. Therefore, using Ag wires as ESDM, in addition to achieving a partial reduction effect, the small piece and dispersed rGO flakes are more independently embedded with Ag nanoparticles (AgNP). Additionally, the composite formed rGOAg has better suspension, conductivity, and antibacterial benefits.

## 2. Materials and Methods 

### 2.1. Experimental System 

**Figure 1:**
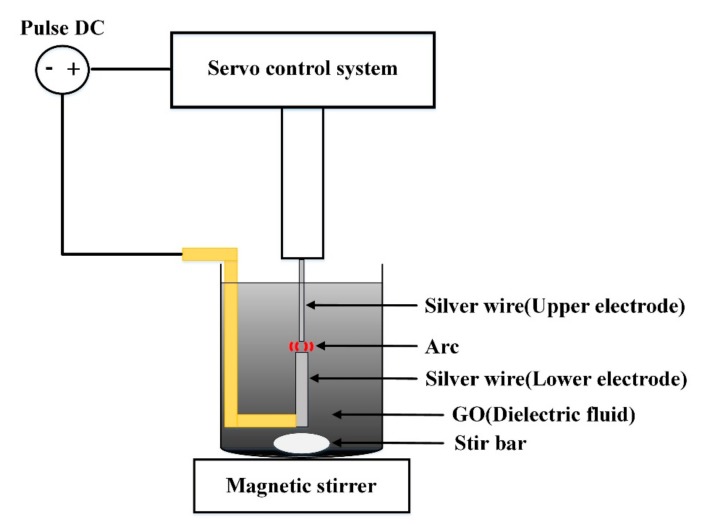
Schematic of rGOAg preparation.

**Figure 2:**
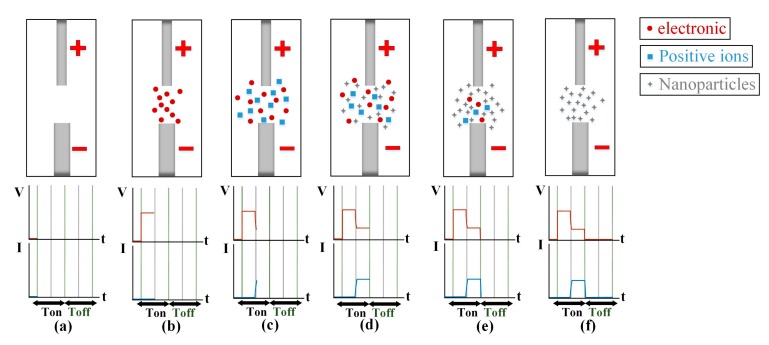
Electrical spark discharge method (ESDM) process: (**a**) prepare to discharge, (**b**) discharge initiation, (**c**) ionization, (**d**) melting effect, (**e**) discharge off, and (**f**) insulation recovery.

This study used the EDM to prepare rGOAg colloids. Figure 1 presents the schematic of the setup. The conducting material was processed using the ESDM, where high heat energy generated using electric energy cuts the conducting material. The conducting material was obtained from the upper and lower electrodes and was immersed in the dielectric fluid with high insulativity. The dielectric fluid could be deionized water (DW) or it could be generated according to experimental requirements. Relevant parameters, such as discharge pulse modulation, current (IP), voltage switching buttons, and capacitance, were set accordingly. Furthermore, a direct current pulse voltage was applied between the two electrodes to form a potential difference, which was termed as a discharge column or arc. Finally, the distance between the electrodes was gradually reduced using a servo control system. When the distance between two electrodes was approximately 30 μm, the highest electric field intensity was observed. The insulativity of the dielectric fluid was damaged, and an arc of 5000–6000 K was formed as in Reference [34]. The high temperature of the arc melted the electrode surface. The high-strength and high-hardness metal materials were ablated [35], and fine metal particles were stripped out and rapidly cooled to form metal nanoparticle (NP). When the discharge pulse ended and insulation was recovered, the ESDM process waited for the next discharge pulse [36]. In the ESDM, the aforementioned steps were repeatedly performed to complete electric conduction. Figure 2 shows the ideal ESDM process, where the term “electronic” represents the electron current. The electron current flows from a cathode to an anode, which represents the direction of electron migration. The process steps were as follows: (a) Prepare to discharge: power is not on, and thus, no voltage or current is present, and the dielectric fluid remains in a state of insulation. (b) Discharge initiation: the electric field gradient of a salient point on the surface of the two electrodes exceeds the withstanding insulation voltage of the dielectric fluid, and numerous electrons are shot from the cathode into the anode, thereby conducting to form an arc. (c) Ionization: when the interpolar state of insulation is damaged, electrons start from the negative electrode and impinge on the interelectrode neutral atoms midway in the dielectric fluid. The outermost valence electron of the atom is excited and forms a cation. The electrons rapidly flow toward the anode, and ionization is performed again to form an ionization channel. The current increases to generate a gap current. (d) Melting effect: when the positive and negative ions impinge on the metal surface, kinetic energy is converted into heat energy to form an electrical discharge arc. The high temperature exfoliates and melts the surface metal, and the electrode metal surface splashes metal particles. (e) Discharge off: the electrode stops releasing electrons, and the current starts to decrease. The ionization channel disappears, and metal particles and metal ions are suspended in the dielectric fluid. The gap voltage and current are decreased. (f) Insulation recovery: the electrode metal surface temperature is reduced, and interelectrode insulation is recovered. The EDM process is completed, and these steps are repeated for the next cyclic discharge.

### 2.2. Dielectric Fluid and Material

The dielectric fluid was obtained through GO dispersion generated by dissolving GO using the Hummers method as described in Reference [37] in DW. A 99.9% pure silver wire was used as an electrode material. For a smooth discharge process, the wire thicknesses for the lower and upper electrodes were 1 and 2 mm, respectively. Therefore, the upper and lower electrodes could be easily aligned in the black GO to increase the arcing rate.

### 2.3. Principle of Reduction

**Figure 3:**
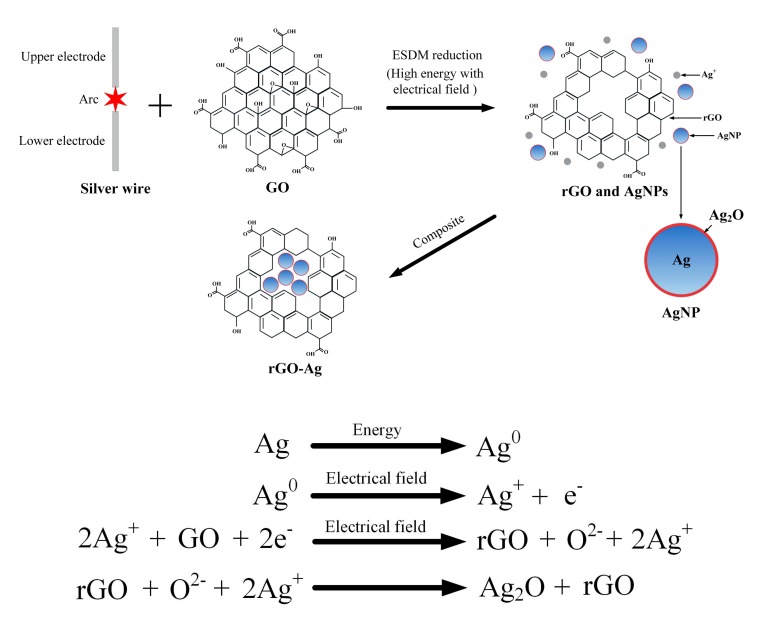
Reduction–oxidation flowchart and chemical equations.

Figure 3 shows the preparation of AgNP through the ESDM using the oxidation–reduction flowchart and chemical equation. The silver ions generated using the Ag electrode discharge had a high electronegativity voltage difference for the oxygen atom, and thus, the atom was carried away from GO, and GO was reduced to rGO. During the discharge process, the electrode gap generates high energy because of high temperature, and thus, the liquid Ag exfoliated from the Ag wire is vaporized to form Ag atoms. Moreover, a high electric field was observed in the electrode zone of arc discharge, and the Ag atoms in the high electric field generated silver ions, electrons, AgNP, and Ag_2_O through continuous ionization. The dielectric fluid used in this study was GO, which has numerous oxygen-containing functional groups, and thus, during preparation, the high temperature and charged Ag atoms of the Ag plasma exhibited a reducing property. The oxygen-containing functional groups in GO were carried away by Ag, wherein GO lost deoxidization functional groups and was reduced to rGO. At this point, Ag_2_O played an intermediate role. Ag_2_O in Ag coated the surface of the AgNP and O attracted the rGO, and thus, the AgNP was encapsulated in rGO, and the rGOAg complex was finally formed.

### 2.4. Experimental Method

This study employed an EDM to prepare metal NPs under crucial environmental parameter settings (Table 1). For rGOAg preparation, 150 mL of GO dispersion in water was poured into a beaker. Silver wires were used as the upper and lower electrodes, and a magnet was rotated in the beaker for the even dispersion of AgNP. The range of T_on_ and T_off_ was 0–999 μs, and the T_on_ and T_off_ were set at 30 μs (a duty cycle of 50%). The IP had seven segments, and the 7th segment (IP = 7) was selected. The total discharge time was 2 min, and nanocolloids were produced at normal temperature and pressure. The voltage switching button and capacitor settings were not required, and thus, AgNP were prepared in GO after the required settings were achieved. GO was reduced, and the AgNP was combined to form rGOAg.

The preparation of AgNP in GO was completed after the complete discharge, and GO was reduced through silver electrode discharge to form the rGOAg complex. This study used ultraviolet-visible spectroscopy (UV-Vis) [38] and electrophoresis [39]. The crystal structure and orientation were evaluated through X-ray diffraction (XRD), and the size and distribution of AgNP in rGOAg were observed through transmission electron microscopy (TEM). The defect level was tested using surface-enhanced Raman spectroscopy (SERS). The surface functional groups were evaluated through Fourier-transform infrared spectroscopy (FTIR), and the surfaces were analyzed through X-ray photoelectron spectroscopy (XPS).

## 3. Results

### 3.1. Suspension Stability

**Figure 4:**
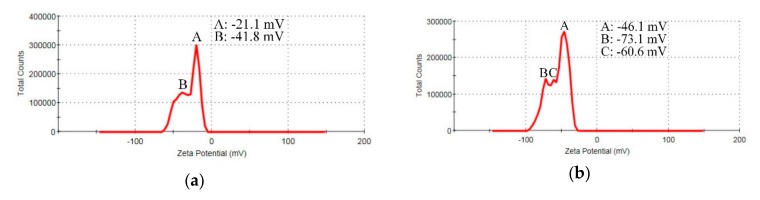
Zeta potential of (**a**) GO; (**b**) rGOAg.

Figure 4 shows the zeta potential results of GO and rGOAg. The calculation result provided the average value of the products of different peaks and percentages. Figure 4a shows GO, where the zeta potential value was −30 mV (Equation (1)); thereby indicating that GO had a high dispersion. GO is difficult to precipitate, has a suitable suspensibility, and has appropriate hydrophilicity. Figure 4b shows the rGOAg colloid, where the zeta potential was −55.5 mV (Equation (2)). The electrophoretic mobility (U_E_) was calculated using the Henry equation (Equation (3)), where U_E_, z, ε, η, and f(ka) were electrophoretic mobility, zeta potential, dielectric constant, viscosity, and the Henry’s function, respectively. The zeta potential and electrophoretic mobility were proportional to viscosity. With the decreasing particle size, the number of particles increased, and thus, the number of interactive particles and viscosity increased. Under the same controlled conditions of electrophoresis, with smaller particles, the electrophoretic mobility was higher. Therefore, during reduction, the quantity of O in rGOAg and surface charge decreased. However, the size of rGOAg decreased, and thus, the viscosity and electrophoretic mobility affected the increase in zeta potential, and the zeta potential and suspensibility increased. The results show that rGOAg has better suspension than GO.

(1) $$\frac{\left(-21.1\right)\times 56.9\%+\left(-41.1\right)\times 43.1\%}{100\%}=-30\;\mathrm{mV}$$

(2) $$\frac{\left(-46.1\right)\times 55.7\%+\left(-73.1\right)\times 27.8\%+\left(-60.6\right)\times 16.6\%}{100\%}=-55.5\;\mathrm{mV}$$

(3) $$U_{E}=\frac{2\varepsilon z}{3\eta }f\left(\mathrm{ka}\right)$$

### 3.2. Characterization of rGOAg

**Figure 5:**
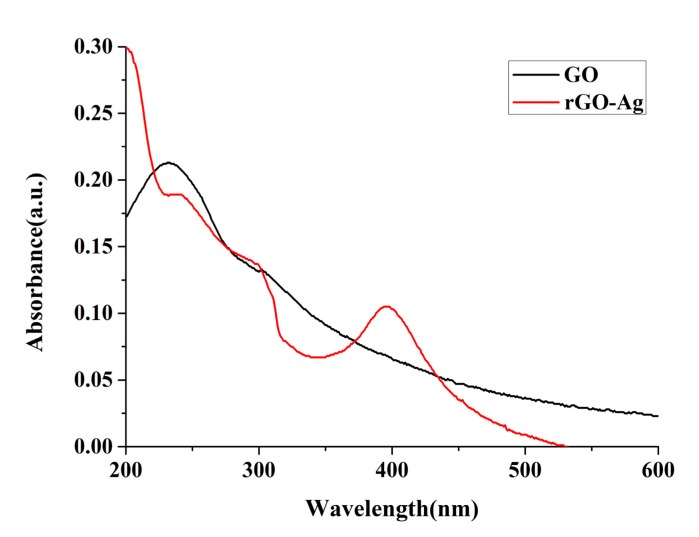
The ultraviolet-visible spectroscopy (UV-Vis) spectra.

Figure 5 shows the UV-Vis spectra. GO has evident absorption peaks at 232 nm. This indicates the π–π transitions of the aromatic C–C bonds, thus validating the presence of GO. rGOAg exhibited different results, and the absorption peak moved from 232 nm to 242 nm. This absorption peak indicated the formation of rGO and the complete reduction of GO. In addition to the absorption peak at 242 nm, rGOAg had an absorption peak at 394 nm, which matched the wavelength range of the AgNP, and resulted from surface plasmon resonance [40]. This phenomenon validated that when GO was used as a dielectric fluid, it was reduced to rGO, and the AgNP was successfully prepared through the ESDM to form the rGOAg complex.

**Figure 6:**
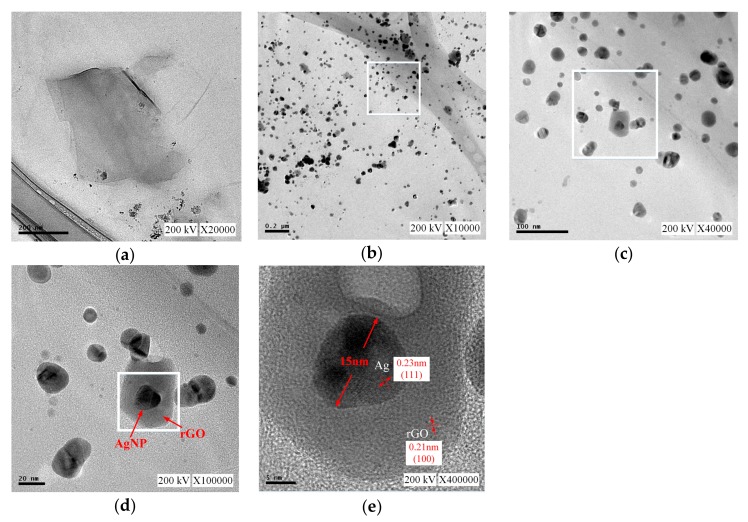
Transmission electron microscopy (TEM) images: (**a**) 200 nm, GO; (**b**) 0.2μm, rGOAg; (**c**) 100 nm, rGOAg; (**d**) 20 nm, rGOAg; and (**e**) 5 nm, rGOAg.

Figure 6 shows the TEM results. Figure 6a shows a scale of 200 nm and a complete flake-like GO, and the size of GO was approximately 300 nm. Figure 6b showed rGOAg, the same scale as that observed in Figure 6a. The original complete GO was reduced to small pieces of rGO after discharge, and the size was approximately 30 nm. Figure 6c shows rGO using a circular gray part [0.21 nm of Figure 6e], and a slight aggregation could be observed, which indicated the high dispersion of rGOAg. The scale in Figure 6d was 20 nm, and the black particles in rGO were AgNP because each AgNP exfoliated from the Ag electrode through the ESDM was coated with rGO, thereby resulting in recombination. In the structure, the AgNP was embedded in the rGO flakes, thus forming the rGOAg complex. The scale of Figure 6e was 5 nm. The size of the AgNP was approximately 15 nm, and the lattice width was 0.23 nm, with a crystal structure of (111). Moreover, the thickness of rGO was 0.21 nm, with a crystal structure of (100) [41].

**Figure 7:**
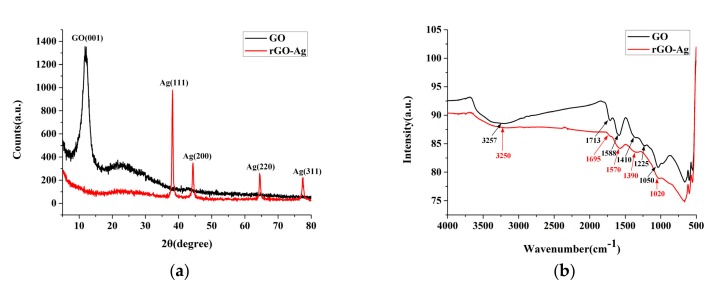
GO and rGOAg: (**a**) X-ray diffraction (XRD) and (**b**) Fourier-transform infrared spectroscopy (FTIR).

Figure 7a shows the XRD patterns. The diffraction peak (JCPDS:01-074-2329) of GO was at 13.6°, and the interlayer spacing was 0.64 nm, with the crystal structure of (001). According to the XRD patterns of rGOAg, the diffraction peak of GO disappeared completely, and most interlayer oxygen functional groups were removed, whilst AgNP and a few oxygen functional groups were retained. Furthermore, apart from the characteristic peak of AgNP, a weak peak was observed at 23.1° when GO was reduced to rGO during the discharge process, which indicated that the original molecular structure of rGO had changed. Moreover, rGO was coated with Ag to form a sphere. rGO was not an ordinary flake and it exhibited a lower sensitivity to XRD than Ag. The characteristic peak strength of rGO was very low. The four diffraction peaks (JCPDS:01-089-3722) of rGOAg were at 38.1°, 44.3°, 64.4°, and 77.4°, and the corresponding crystal structures were (111), (200), (220), and (311). This observation validates the presence of AgNP in the form of crystals in rGOAg [42,43]. Figure 7b shows the FTIR spectra. The O-H stretching was observed at 3257 and 3250 cm^−1^, and C=O stretching was observed at 1713 and 1695 cm^−1^. C=C bonding of the GO carbon skeleton was observed at 1588 and 1570 cm^−1^. O–H deformation was observed at 1410 and 1390 cm^−1^. C–OH stretching was observed at 1225 cm^−1^, whereas C-O stretching was observed at 1050 and 1020 cm^−1^. The characteristic peaks of rGOAg were close to the peaks of GO. Therefore, the characteristic peak relative strength of rGOAg oxygen-containing functional groups degraded [44]. These results showed that a part of the oxygen-containing functional groups in GO was removed, thereby validating that GO could be reduced to the rGOAg complex using the ESDM and silver electrode.

**Figure 8:**
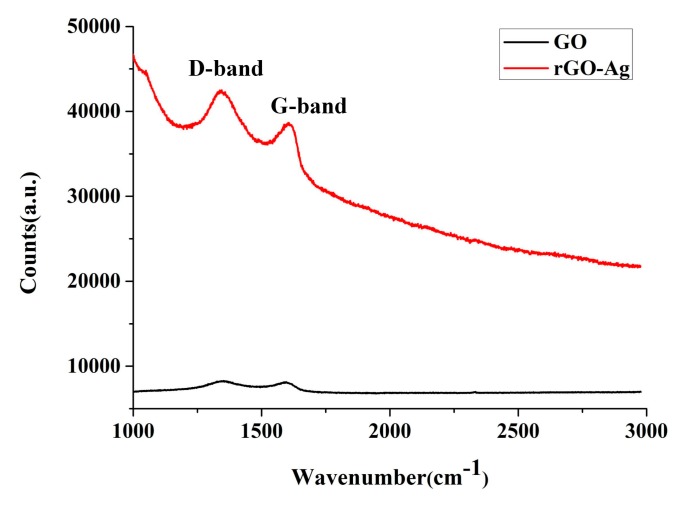
The Raman spectra.

Figure 8 shows the Raman spectra. GO and rGOAg exhibited two graphene peaks at the D-band and G-band. The D-band was a disordered vibration peak and represented the defect and breakage of graphene. The G-band was the primary characteristic peak of the sp^2^ carbon atom. The D-band and G-band peaks were observed near 1350 and 1580 cm^−1^, respectively. The defect level was calculated based on the intensity ratio (I_D_/I_G_) of the D-band and G-band. The ratios of GO and rGOAg were 1.03 and 1.07, respectively. Therefore, rGOAg had a higher defect level than GO [45]. Since partial oxygen-containing functional groups were removed, rGOAg may have a considerable amount of defective bonding and compound Ag^+^. SERS revealed evident effects in the Raman spectra of GO and rGOAg. The D-band and G-band Raman signal strengths of rGOAg increased, thus validating that the rGOAg complex as prepared by ESDM could enhance the Raman signal strength of GO [46].

**Figure 9:**
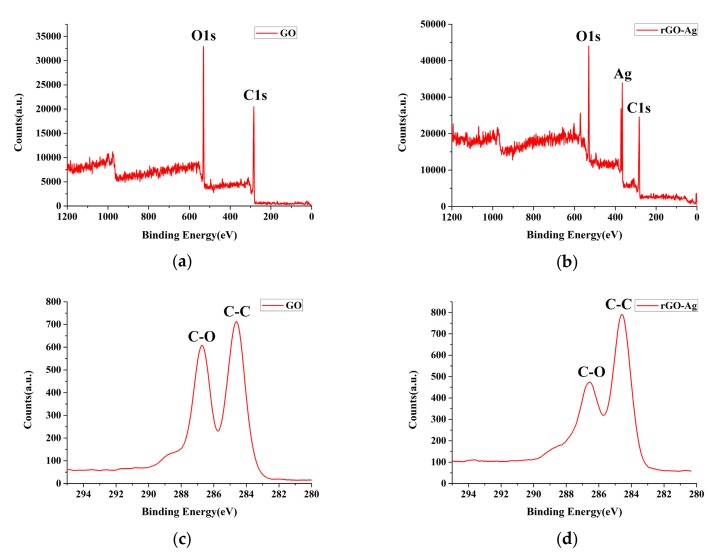
X-ray photoelectron spectroscopy (XPS) spectrum (**a**) survey spectrum of GO; (**b**) survey spectrum of rGOAg; (**c**) C1s spectrum of GO; (**d**) C1s spectrum of rGOAg.

Figure 9 shows the XPS spectra. However, because ESDM mainly removed O^2−^, OH^−^ and COOH^−^ remained in the edges of the rGO structure, minimally changing the C/O ratios. The XPS spectrum of GO (0–1200 eV) had two peaks at the C1s band (284.6 eV) and O1s band (531.0 eV). Figure 9b shows the XPS spectrum of rGOAg (0–1200 eV). In addition to the C1s and O1s bands, the Ag band (367.0 eV) was detected, thus validating the presence of Ag. Figure 9c,d show the C1s spectra of GO (280–294 eV) and rGOAg (280–294 eV), where two peaks were observed at 284.6 and 286.5 eV, which were C–C and C–O bands of the graphene material, respectively [47]. Compared to the peak intensities of the C–C and C–O bands of GO, the peak intensity of rGOAg decreased, that is, a part of the oxygen-containing functional groups was successfully removed after the EDM discharge, and reduction occurred.

## 4. Discussion

This study used the EDM to perform the ESDM and used an Ag electrode to discharge in GO for reducing GO to form an rGOAg complex at normal temperature and pressure, without using any chemical substances. The contributions of this study are as follows:

1. This new reduction method is a simple process with a short preparation time. This environment-friendly process does not require additional chemical substances.
2. According to UV-Vis, XRD, FTIR, and XPS, after GO and the Ag electrode were processed through the ESDM, both AgNP and Ag^+^ ions were generated, the latter of which was generated via Ag plasma, because the charged Ag atoms of Ag plasma had a strong reducing property, GO was reduced to the rGOAg complex.
3. According to the analysis of Zetasizer, the rGOAg complex has a larger zeta potential than GO. Since Ag plasma pyrolyzed GO bulk into GO flakes and generated Ag^+^ ions during the process, the strong reducing effect stripped away O between the GO bulk layers. Subsequently, rGO flakes covered the AgNP, forming an rGOAg complex with rGO flakes that possessed sufficient functional groups and H_2_O in the edges. The functional groups and H_2_O then formed hydrogen bonds, which elevated the suspension and dispersibility of the rGOAg complex.
4. The TEM analysis showed that the complete GO was broken into small pieces of rGO flakes, and the AgNP were embedded in rGO flakes with decorated surface function groups. The suspensibility and dispersion of AgNP were enhanced, and rGO coated on the AgNP surface was hydrophobic. In medical treatments, rGOAg is more likely to penetrate through the bacterial cell membrane, thus improving antibacterial ability.

## 5. Conclusions

The ESDM is a physical method for preparing NPs, and in contrast to the chemical method, it is free from any chemical substances and can be performed at normal temperature and pressure. Furthermore, the NPs with high suspensibility can be prepared in a short time. Ag metal was used as a discharge electrode, and GO was successfully reduced to an rGOAg complex through the ESDM. The surface of AgNPs was coated with rGO flakes. The suspensibility and dispersion of rGOAg were improved. These results indicated that the proposed method could be used for reducing GO to rGOAg.

## Figures and Tables

**Table 1:** Experimental parameters for using an electrical discharge machine (EDM) to prepare rGOAg.

Pulse Discharge Cycle (T_on_:T_off_)	Dielectric Fluid	Electrode	Discharge Time	Voltage
30:30 us	GO dispersion in water	Ag	2 min	140 V
**Atmospheric pressure**	**Volume of the dielectric fluid**	**Purity of the electrode**	**Diameter of the electrode (Upper/Lower)**	**Current segment setting**
1 atm	150 mL	99.99%	1/2 mm	7 IP
